# Chemoselective *O*-acylation of hydroxyamino acids and amino alcohols under acidic reaction conditions: History, scope and applications

**DOI:** 10.3762/bjoc.11.51

**Published:** 2015-04-08

**Authors:** Tor E Kristensen

**Affiliations:** 1 Land Systems Division, Norwegian Defence Research Establishment (FFI), P.O. Box 25, NO-2027 Kjeller, Norway

**Keywords:** amino alcohols, chemoselectivity, DOPA, hydroxyamino acids, hydroxyproline, *O*-acylation, organocatalysis, serine, threonine, tyrosine

## Abstract

Amino acids, whether natural, semisynthetic or synthetic, are among the most important and useful chiral building blocks available for organic chemical synthesis. In principle, they can function as inexpensive, chiral and densely functionalized starting materials. On the other hand, the use of amino acid starting materials routinely necessitates protective group chemistry, and in reality, large-scale preparations of even the simplest side-chain derivatives of many amino acids often become annoyingly strenuous due to the necessity of employing protecting groups, on one or more of the amino acid functionalities, during the synthetic sequence. However, in the case of hydroxyamino acids such as hydroxyproline, serine, threonine, tyrosine and 3,4-dihydroxyphenylalanine (DOPA), many *O*-acyl side-chain derivatives are directly accessible via a particularly expedient and scalable method not commonly applied until recently. Direct acylation of unprotected hydroxyamino acids with acyl halides or carboxylic anhydrides under appropriately acidic reaction conditions renders possible chemoselective *O*-acylation, furnishing the corresponding side-chain esters directly, on multigram-scale, in a single step, and without chromatographic purification. Assuming a certain degree of stability under acidic reaction conditions, the method is also applicable for a number of related compounds, such as various amino alcohols and the thiol-functional amino acid cysteine. While the basic methodology underlying this approach has been known for decades, it has evolved through recent developments connected to amino acid-derived chiral organocatalysts to become a more widely recognized procedure for large-scale preparation of many useful side-chain derivatives of hydroxyamino acids and related compounds. Such derivatives are useful in peptide chemistry and drug development, as amino acid amphiphiles for asymmetric catalysis, and as amino acid acrylic precursors for preparation of catalytically active macromolecular networks in the form of soluble polymers, crosslinked polymer beads or nanoparticulate systems. The objective of the present review is to increase awareness of the existence and convenience of this methodology, assess its competitiveness compared to newer and more elaborate procedures for chemoselective *O*-acylation reactions, spur its further development, and finally to chronicle the informative, but poorly documented history of its development.

## Introduction

Any adept researcher within the field of organic synthetic chemistry will be mindful of the outstanding importance of amino acids as inexpensive chiral starting materials in the synthesis of a nearly infinite variety of synthetic end products. This review is not the place to expound on the numerous facets of amino acid chemistry, but useful references can be consulted as a starting point [1–2]. However, many researchers will also be equally wary of certain inconveniences regularly associated with the use of densely functionalized amino acids as starting materials for synthesis. For example, side-chain chemical manipulation of the most common α-amino acids is routinely preceded by protection of one or both of the amino acid functionalities. In addition to lengthening the synthetic sequence by adding auxiliary steps, protective groups for amino acids can also impart a reduced tendency for crystallinity in the resulting amino acid derivatives, prohibiting, or at least, severely restricting the use of convenient recrystallization for purification on a multigram-scale.

In the case of hydroxy-α-amino acids, any side-chain acylation will, often for no better reason than intuitive inclination or accustomed habit, commonly direct an organic chemist to adopt the ubiquitous Schotten–Baumann-type alkaline acylation conditions (albeit not necessarily the aqueous conditions used in the original procedure) [3], both for attachment of prerequisite protective groups at the amino and carboxy termini of the amino acid (resulting in carbamate and/or ester protective groups) [4], as well as for the subsequent side-chain acylation. Relevant examples are too numerous to render possible any exhaustive or useful listing herein, but use of modern computerized structure-adapted database searches will uncover such protected amino acid derivatives in abundance, with little effort involved. Part of the reason for the impulsive willingness to adopt protective group chemistry is most surely connected to the fact that only minor quantities of end material are required for many applications, particularly within the academic sector. Purification by flash column chromatography then easily becomes a practical, standardized procedure.

If a longer historical perspective is adopted, dating prior to the advent of widespread flash column chromatography, one will realize that amino acid chemistry has fostered the development of a number of specialized methodologies to handle the manifold functional groups present in the most common amino acids. A salient example is the copper chelation chemistry originally introduced by Kurtz in 1941 to isolate lysine from protein hydrolysates [5]. Conversion of amino acids to their copper salts elegantly masks both amino acid functionalities, thus protecting them during a subsequent alkaline acylation reaction that targets the side-chain functionality selectively. The side-chain-acylated lysine can then be liberated by decomposition of the copper salt with hydrogen sulfide. This methodology has subsequently been employed in a more generic sense as a preparative method for synthesis of acrylic side-chain derivatives of amino acids such as lysine, ornithine, tyrosine and serine [6–8], having modified the original technique by liberating the acylated derivative from its copper salt using 8-hydroxyquinoline as an organic chelating precipitant [8].

While both useful and creative, the copper chelation procedure carries with it many of the same shortcomings as other types of protective group chemistry. However, by a change of the conventional mindset, embracing acidic, instead of alkaline acylation conditions, a potentially nimble and direct conversion of unprotected hydroxyamino acids into their corresponding side-chain esters, in a single step, can be envisaged. In a suitable acidic medium, amino groups will be prevented from acylation by protonation (supressing amide formation) and carboxyl acylation to form anhydrides will be minimized by keeping the carboxylic acid strictly in its non-dissociated form. Acylation with acyl halides or carboxylic anhydrides under such conditions would therefore favour side-chain ester formation, thus enabling direct chemoselective conversion of the hydroxyamino acid to the *O*-acyl derivative. Precipitation of the reaction product as a (preferably) crystalline amine salt would further aid the convenience and attractiveness of this methodology. But, such methodology has in fact existed for a considerable period of time, having its origins in biochemical studies, but has not become widespread until recent developments related to organocatalysis further improved and popularized its application. Hopefully, the present work will convince chemists unfamiliar with the procedure how it in many cases can be distinctly preferable to other, including newer and more widely publicized, preparatory strategies for chemical manipulation of amino alcohols and other functionally related substances.

## Review

### The historical development of acidic chemoselective *O*-acylation procedures for hydroxyamino acids

The historical development of acidic chemoselective *O*-acylation procedures for hydroxyamino acids follows no clear or coherent pathway. It appears as a fragmented and loosely connected assemblage of individual reports merely related by their common use of acidic acylation conditions. This loose coherence has perhaps denied these procedures an appearance of all belonging to a singular methodology. The reason for this may be connected to the fact that the fundamental challenge in the development of a broadly useful, generic *O*-acylation procedure for hydroxyamino acids or amino alcohols was dependent on the identification of a reaction medium that satisfied a number of essential criteria: 1) It must be sufficiently acidic to ensure complete protonation of amine functionalities during reaction, 2) it should not have oxidizing properties that can consume the organic substrate, 3) it must dissolve the (generally) very poorly soluble hydroxyamino acids efficiently, 4) it should preferably be fully miscible with a wide range of acyl halides and carboxylic anhydrides (including long-chained fatty-acid derived ones) and if possible, 5) the acid constituent(s) should have only a moderate affinity for amines such that crystalline hydrochloride salts may be precipitated directly from the medium in acylation reactions employing acyl chlorides. The eventual identification of anhydrous trifluoroacetic acid (CF_3_CO_2_H) as perhaps the most appropriate reaction medium identified so far for such acidic acylation reactions will therefore be an important theme throughout most of this text. However, this methodology is founded upon a few pioneering but more narrow-scoped precursor techniques.

#### Early chemoselective *O*-acetylation of hydroxyamino acids

**Scheme 1:**
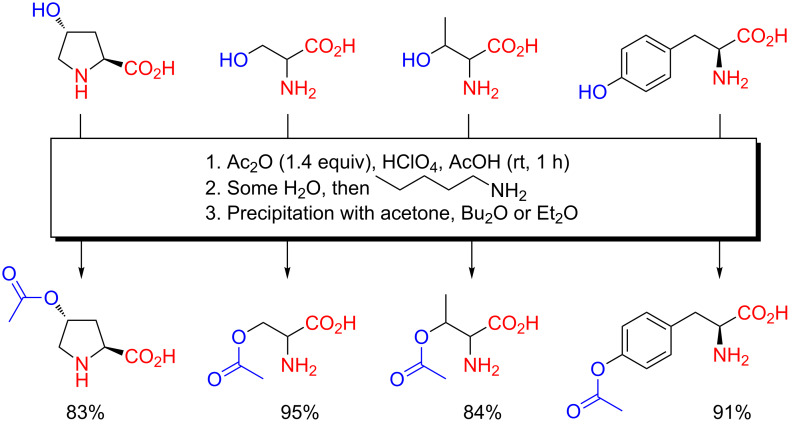
Selective *O*-acetylation of hydroxyamino acids with acetic anhydride in perchloric acid-acetic acid as reported by Sakami and Toennies in 1942 [9–10]. The same method has later been used by Frankel, Cordova and Breuer for DL-serine in 1953 [11], and by Kurtz, Fasman, Berger and Katchalski for hydroxy-L-proline in 1958 [12]. Others have since followed suit.

In 1942, Sakami and Toennies reported a method for the preparation of the *O*-acetyl derivatives of naturally occurring hydroxyamino acids (Scheme 1), a method that was also patented [9–10]. Being mindful of the known accelerating effect of the presence of acids in acetylation reactions of alcohols with acetic anhydride as well as the inhibitory effect of strong acids on the acetylation of amines with acetic anhydride, Sakami and Toennies scrutinized this effect through use of perchloric acid (HClO_4_), at first during titration experiments involving the acetylation of certain amino acids. They then exploited this reactivity pattern to develop a preparatory method for the class of *O*-acetylhydroxyamino acids. They were in fact the first researchers to at all investigate compounds belonging to this class of amino acid derivatives. Under the mantra «acidity favors *O*-acylation, while alkalinity favors *N*-acylation», they accomplished this feat by treatment of hydroxy-L-proline, DL-serine, DL-threonine or L-tyrosine with a solution prepared from acetic anhydride, concentrated aqueous HClO_4_ and glacial acetic acid (excess acetic anhydride ensured consumption of water and formation of anhydrous conditions prior to acetylation) [9–10]. The remaining acetic anhydride was quenched with water after acetylation, and the product in salt-free form was obtained by addition of amylamine and a suitable precipitating liquid (acetone, Et_2_O, Bu_2_O).

The thorough study of Sakami and Toennies neatly summarizes what had been known until then, regarding the reactivity of hydroxy versus amine functionalities in hydroxyamino acids under acylating conditions. In their landmark study, they noted: “Suitable modifications of our procedure, involving the use of benzoic or other anhydrides and the corresponding or inert solvents, may permit its extension to the preparation of other *O*-acyl derivatives” [9]. Indeed, this would prove to be a somewhat prophetic statement, but such a procedure could not materialize itself until a more suitable reaction medium had been identified.

**Scheme 2:**
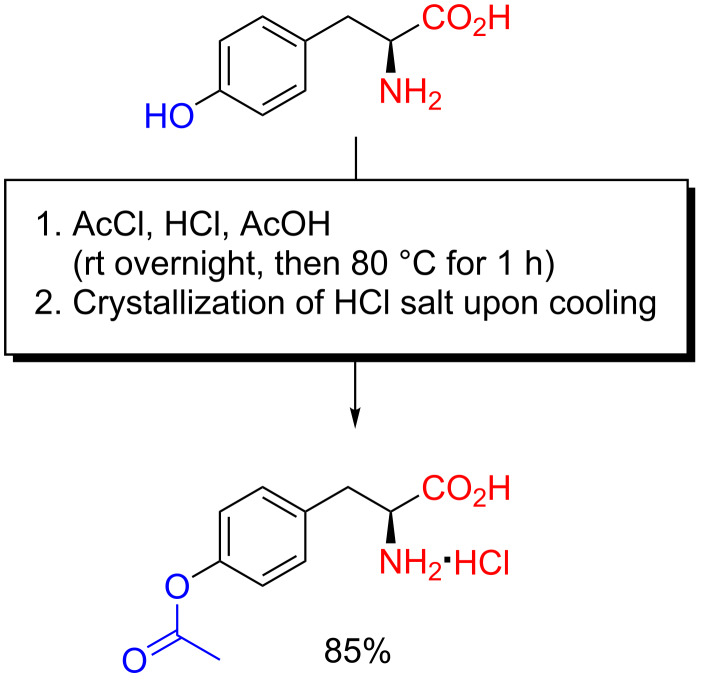
Selective *O*-acetylation of L-tyrosine as reported by Bretschneider and Biemann in 1950 [13].

The chemoselective *O*-acetylation of hydroxyamino acids using acetic anhydride in acetic acid–HClO_4_ as pioneered by Sakami and Toennies was employed by others, as for example in the preparation of *O*-acetyl-DL-serine reported by Frankel, Cordova and Breuer in 1953 [11], and *O*-acetylhydroxy-L-proline by Kurtz, Fasman, Berger and Katchalski in 1958 [12]. However, other and perhaps more ad hoc procedures for the preparation of *O*-acetylhydroxyamino acids emerged next. During the late 1940s, Bretschneider examined the acylation of amino alcohol compounds such as synephrine in much detail (detailed in the later section on amino alcohols). As part of these investigations, and aware of the work Sakami and Toennies [9], he developed acidic *O*-acylation procedures for such substrates, a procedure referred to as salt acylation. In 1950, Bretschneider and Biemann reported a chemoselective *O*-acetylation of L-tyrosine under acidic conditions (Scheme 2) [13].

Acetylation of L-tyrosine with acetyl chloride in HCl-saturated glacial acetic acid gave the *O*-acetylated product upon cooling with ice/NaCl, and unlike the method of Sakami and Toennies, the procedure of Bretschneider and Biemann furnished the product as the hydrochloride salt. This salt could be recrystallized from MeOH–Et_2_O [13]. As an interesting note, in 1953, Schlögl, Wessely and Wawersich mentioned this *O*-acetylation method in particular, as part of their investigations of poly-L-tyrosine, but they rejected it in favour of copper chelate protection since the acidic acylation gave the product in salt form [14]. They also evaluated the method of Sakami and Toennies, but likewise rejected it, much because of its use of expensive amylamine for liberation of the amino acid derivative from its salt. In 1957, Harwood and Cassidy, patterned by the synthesis of polytyrosine by Schlögl, Wessely and Wawersich [14], and polyserine by Frankel, Cordova and Breuer [11], extended the method of Bretschneider and Biemann to the diacetylation of 3,4-dihydroxyphenylalanine (DOPA), opening up for their improved synthesis of poly-DOPA [15].

**Scheme 3:**
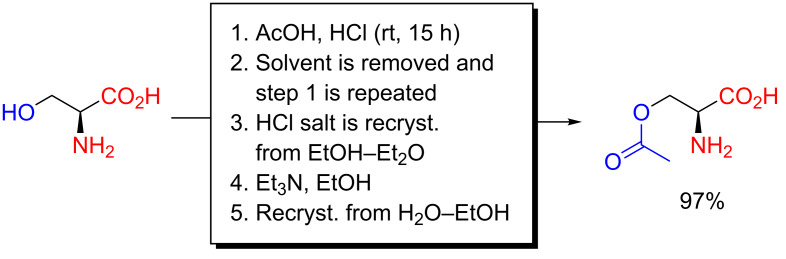
Selective *O*-acetylation of L-serine in acetic acid saturated with hydrogen chloride as reported by Sheehan, Goodman and Hess in 1956 [16]. The same method has been reported by Fasman and Blout for L-serine and DL-serine in 1960 [17], by Arakawa, Smeby and Bumpus for L-serine in 1962 [18] and by Fujiwara, Morinaga and Narita for L-serine, DL-threonine and D-threonine in 1962 [19].

As part of their influential studies on carbodiimide coupling agents for peptide synthesis, Sheehan, Goodman and Hess reported, in 1956, a chemoselective *O*-acetylation of L-serine dissolved in glacial acetic acid saturated with HCl. After storage for 15 h, the solvent was evaporated under reduced pressure and the process was repeated (Scheme 3) [16]. The rather forcing conditions are probably a result of not employing a more reactive acylating agent than acetic acid. The product was obtained as its hydrochloride salt, which was then recrystallized from EtOH–Et_2_O, liberated from its salt using Et_3_N in EtOH and finally recrystallized from aqueous EtOH. Although perhaps a bit tedious, the method nevertheless furnished product at the gram-scale in quantitative yield. Their procedure was also adopted by others, as reported by Fasman and Blout in 1960 for the *O*-acetylation of L-serine and DL-serine [17], by Arakawa, Smeby and Bumpus for L-serine in 1962 [18] and by Fujiwara, Morinaga and Narita in 1962 for L-serine, DL-threonine and D-threonine [19].

**Scheme 4:**
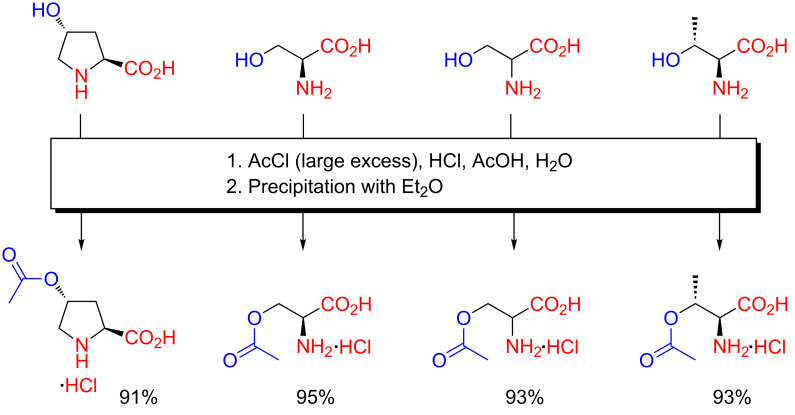
Chemoselective *O*-acetylation of hydroxyamino acids with acetyl chloride in hydrochloric acid–acetic acid as reported by Wilchek and Patchornik in 1964 [20].

Although they are large-scale adaptable, acidic *O*-acetylation reactions using HCl-saturated glacial acetic acid solutions are undeniably somewhat cumbersome due to the necessity of handling gaseous HCl. As a result, into the 1960s, the need for a truly convenient *O*-acetylation procedure for hydroxyamino acids remained unsatisfied. A generalizable *O*-acylation method seemed even more remote. In 1964, Wilchek and Patchornik then reported the first truly convenient procedure for the preparation of *O*-acetylhydroxyamino acids through acidic acetylation (Scheme 4) [20]. The relevant hydroxyamino acid was simply dissolved in a mixture of hydrochloric acid and glacial acetic acid and acetylated in a few minutes by slow addition of a large excess of acetyl chloride at 0 °C. The product was obtained as the crystalline hydrochloride salt through precipitation by addition of Et_2_O. The *O*-acetyl hydrochlorides of hydroxy-L-proline, L-serine, DL-serine and L-threonine were all obtained directly in excellent purity in over 90% yield at >10 g scale.

#### Early *O*-acylation of hydroxyamino acids in trifluoroacetic acid

As narrated in the previous section, developments taking place from the early 1940s into the early 1960s had resulted in several useful techniques for the preparation of *O*-acetyl derivatives of hydroxyamino acids. Unfortunately, none of these could be further developed into a more generalizable *O*-acylation procedure in any straightforward manner. However, developments taking place in the same time period would eventually open up for just such a possibility.

Trifluoroacetic acid (CF_3_CO_2_H) has been known since 1922 [21], but its widespread application was only made possible sometime later, much due to the development of electrochemical fluorination processes (Simons process) for production of organofluorine compounds in the 1930s and 1940s [22–23]. It is a strong acid (p*K*_a_ ≈ 0.5), quite volatile (bp. 72 °C) and miscible with water, fluorocarbons and most organic solvents [22]. In the late 1940s and throughout the 1950s, trifluoroacetic acid and trifluoroacetic anhydride were the subjects of a number of reported chemical studies, probing their characteristics and chemical reactivity. Among these, it was reported in 1951 that a mixture of a carboxylic anhydride and CF_3_CO_2_H was effective for acylation of alcohols [24]. Further on, in 1954, it was reported that anhydrous CF_3_CO_2_H was a powerful solvent for proteins [25]. Combining these two characteristics of CF_3_CO_2_H, Bello and Vinograd then disclosed a method in 1956 for selective acetylation of hydroxy groups in gelatin [26]. In addition to this novel acetylation using acetic anhydride in CF_3_CO_2_H, the older method of Sakami and Toennies (HClO_4_-acetic anhydride in glacial acetic acid) was also tested out. Unfortunately, the researchers did not proceed to develop the results into a generalizable, chemoselective *O*-acylation methodology for hydroxyamino acids and related derivatives.

**Scheme 5:**
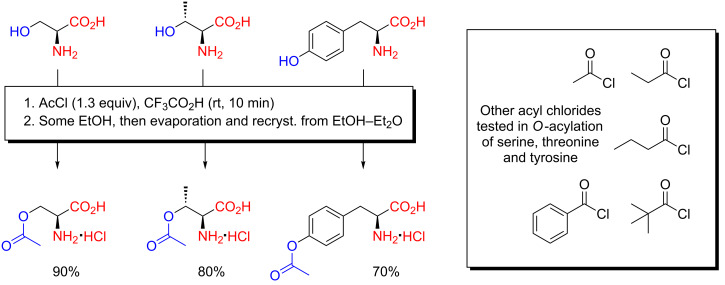
Chemoselective *O*-acylation of hydroxyamino acids with acyl chlorides in anhydrous trifluoroacetic acid as reported by Previero, Barry and Coletti-Previero in 1972 [27]. Cysteine is chemoselectively *S*-acylated under the same conditions.

The decisive breakthrough in the development of a widely applicable chemoselective *O*-acylation methodology for hydroxyamino acids was finally achieved by Previero, Barry and Coletti-Previero in 1972 (Scheme 5), by a method they referred to as specific *O*-acylation of hydroxyamino acids [27]. Hydroxyamino acids were treated with a slight excess of acyl chloride in CF_3_CO_2_H at room temperature, and the product was isolated by evaporation of the solvent and recrystallization of the residue from EtOH–Et_2_O. Because of the non-competitive nucleophilic character of the solvent, a great excess of reagent was unnecessary. Although the authors clearly realized the generally applicable character of their method through the possibility of employing other acyl chlorides than acetyl chloride, this work apparently did not trigger a decisive breakthrough in the widespread application of acidic chemoselective *O*-acylation methodologies in organic synthesis. Although speculative, the origin of the method in biochemical circles, as a result of investigations into protein chemistry in organic solvents (also evidenced in the venue for publication), may have obscured its visibility somewhat to the community of organic chemists. The absence of hydroxyproline is also noticeable.

Instructively, Previero, Barry and Coletti-Previero undertook a comprehensive survey of the stability of the most common amino acids in CF_3_CO_2_H medium as part of their work (Table 1) [27]. The complete recovery of these amino acids is encouraging and a testimony to their inertness under non-nucleophilic, acidic reaction conditions.

**Table 1:** Recovery of amino acids after trifluoroacetic acid–acetyl chloride treatment as reported by Previero, Barry and Coletti-Previero in 1972 [27].

Amino acid	Recovery [%]^a^

Lysine	97
Histidine	99
Arginine	98
Aspartic acid	99
Glutamic acid	96
Glycine	102
Alanine	100
Valine	99
Methionine	97
Isoleucine	101
Leucine	100
Phenylalanine	100
Proline	98
1-Formyltryptophan^b^	98

^a^Recovery after treatment of amino acid in CF_3_CO_2_H solution with 15 equiv of acetyl chloride for 20 min. ^b^Tryptophan is irreversibly modified during *O*-acylation.

Previero, Barry and Coletti-Previero also reported in their work how the *O*-acetyl derivatives of serine and threonine are converted to their corresponding *N*-acetyl derivatives by mild basic treatment [27]. This well-known O→N acyl shift had been described in more detail by others previously [19], and it can be used to separate serine and threonine from mixtures of other amino acids (by acidic *O*-acetylation followed by O→N acetyl shift under basic conditions and subsequent separation of the *N*-acetyl derivatives of serine/threonine from other amino acids with ion exchange resins) [19,27]. Therefore, *O*-acyl derivatives of hydroxyamino acids prepared by chemoselective *O*-acylation can advantageously be stored and handled as the "blocked" salts prior to use, in order to avoid such an O→N acyl shift.

The use of CF_3_CO_2_H in acidic acylations is associated with a particular advantage. Its low affinity for amines indicates that the acylation products normally can be isolated as their crystalline hydrochloride salts from the reaction mixture, provided that an acyl chloride has been used as the acylating reagent. Previero, Barry and Coletti-Previero do not seem to have recognized and exploited this useful feature of CF_3_CO_2_H as acylation medium, instead relying on evaporation of the reaction mixture. This will be an important point in later sections.

#### Chemoselective *O*-acylation of hydroxyamino acids in methanesulfonic acid or acidified organic solvents

As stated previously, the disclosure of trifluoroacetic acid as an excellent medium for acidic *O*-acylation of hydroxyamino acids in the early 1970s apparently did not induce it to become a dominating method for chemoselective *O*-acylations, at least for the time being. Instead, during the 1980s, an alternative medium for acidic *O*-acylation of hydroxyamino acids in the form of methanesulfonic acid (MeSO_3_H) was identified.

In 1977, chemists in India had reported that L-tyrosine, hydroxy-L-proline, L-serine, DL-serine and DL-threonine could all be chemoselectively *O*-acetylated overnight in high chemical yields with acetic anhydride in glacial acetic acid containing an excess of sulfonic acid polystyrene resin [28]. The use of a polymeric resin also facilitated isolation of the reaction products, but it limited the scale of reaction because of the low capacity of the resin (but the reactions were nonetheless undertaken at gram-scale). Again, although innovative, the method cannot be easily generalized to other *O*-acylations. However, the use of sulfonic acids in acidic *O*-acylations was instructive [29–30].

**Scheme 6:**
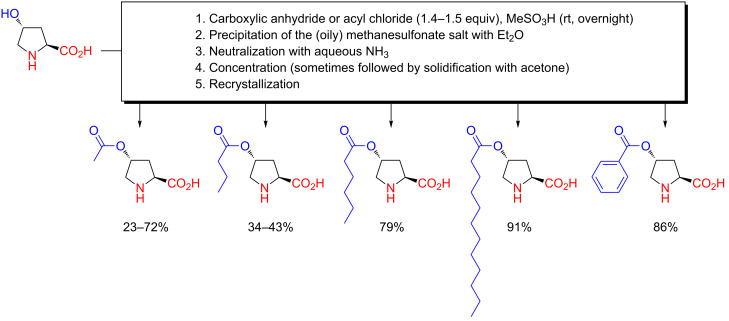
Chemoselective *O*-acylation of hydroxyproline with acyl chlorides or carboxylic anhydrides in methanesulfonic acid as reported by Kawasaki and Komai in 1983 [34].

During the years 1979 to 1981, a number of Japanese scientists reported on chemical modification of chitin (a naturally occurring polymer of *N*-acetylglucosamine) by *O*-acylation, using carboxylic anhydrides or acyl chlorides in methanesulfonic acid solution [31–33]. Taking inspiration from these disclosures, Kawasaki and Komai then, in 1983, reported on the chemoselective *O*-acylation of hydroxyproline with carboxylic anhydrides or acyl chlorides in MeSO_3_H (Scheme 6) [34]. After acylation of hydroxyproline at the >10 g scale in MeSO_3_H overnight at room temperature, the product was separated as the (usually oily) methanesulfonate salt by addition of Et_2_O. The salts were neutralized with aqueous NH_3_, and the free *O*-acylhydroxyamino acids were recrystallized from suitable solvents such as acetone, lower alcohols and H_2_O.

Acylation reactions in MeSO_3_H are more vigorous and exothermic than in CF_3_CO_2_H since MeSO_3_H (p*K*_a_ ≈ −2) is a stronger acid than CF_3_CO_2_H (p*K*_a_ ≈ 0.5). Their densities are the same (1.48 g/mL at 25 °C), but the melting point of MeSO_3_H (20 °C) is considerably higher than that of CF_3_CO_2_H (−15 °C), and the volatility of MeSO_3_H is insignificant compared to CF_3_CO_2_H, making evaporation of reaction mixtures based on MeSO_3_H entirely unfeasible [22,29–30]. Unlike CF_3_CO_2_H, MeSO_3_H has a higher affinity for amines than does HCl, making the crystalline hydrochlorides unavailable from MeSO_3_H medium, thereby complicating the isolation and purification process due to the poor crystallinity of methanesulfonate salts, which in fact, quite frequently are ionic liquids. On the positive side, MeSO_3_H is biodegradable.

In addition to the relatively broadly applicable and chemoselective *O*-acylation procedures in CF_3_CO_2_H or MeSO_3_H just mentioned, a number of reports have surfaced from the 1940s and up to the present on chemoselective *O*-acylation of various amino alcohols in organic solvents under acidic conditions. A more detailed account of some of these disclosures will be postponed to a later section, where they will be combined with others into an overall evaluation of the use of acidic conditions for chemoselective *O*-acylation of amino alcohol structural motifs.

**Scheme 7:**
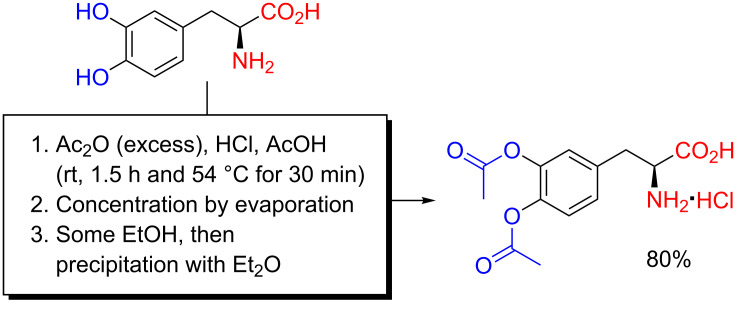
Chemoselective *O*-acetylation of L-DOPA as reported by Fuller, Verlander and Goodman in 1978 [35].

Besides CF_3_CO_2_H and MeSO_3_H media, acidic *O*-acylation of hydroxyamino acids in acidified organic solvents has been reported on several occasions. It has been especially prevalent for tyrosine and DOPA, the latter being the biologically important precursor to the catecholamine neurotransmitters. As for acetylation of DOPA, Fuller, Verlander and Goodman reported, in 1978, a method much akin to that by Harwood and Cassidy from 1957 for diacetylation of L-DOPA (Scheme 7), although using acetic anhydride instead of acetyl chloride [15,35].

A suspension of L-DOPA in glacial acetic acid was saturated with dry HCl gas, and acetic anhydride was added. After continued purging with HCl, addition of more acetic anhydride and heating of the reaction mixture to 54 °C for 30 min, the mixture was subsequently concentrated under reduced pressure, quenched with EtOH, and the product was isolated at the >10 g scale, as the hydrochloride salt, by precipitation with Et_2_O. Such *O*,*O'*-diacetyl L-DOPA has since been used by many others as a protected L-DOPA derivative, useful for further synthetic manipulations, such as in biology-oriented materials science and drug delivery.

**Scheme 8:**
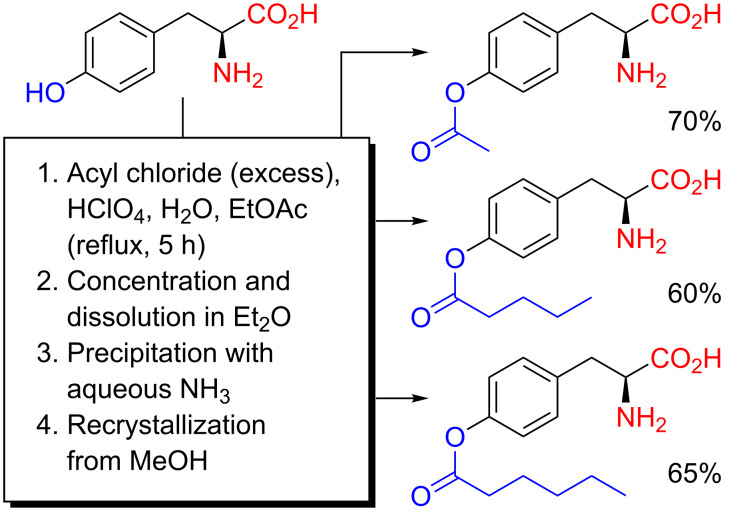
Chemoselective *O*-acylation of L-tyrosine as reported by Huang, Kimura, Bawarshi-Nassar and Hussain in 1985 [36].

Methods for *O*-acylation taking place in acetic acid are limited to acetylation, but use of inert organic solvents allows for an extension to other acyl moieties. In 1985, Huang, Kimura, Bawarshi-Nassar and Hussain reported *O*-acylations of L-tyrosine in ethyl acetate acidified with aqueous HClO_4_ [36]. A solution of L-tyrosine in a mixture of HClO_4_ (70%) and EtOAc was acylated with acyl chlorides at reflux. Isolation, neutralization with aqueous NH_3_ and recrystallization from MeOH then furnished *O*-acetyl-L-tyrosine, *O*-valeryl-L-tyrosine and *O*-hexanoyl-L-tyrosine at the gram-scale (Scheme 8). It should be noted that the use of concentrated HClO_4_ in an organic solvent, at reflux temperatures, may be a bit precarious, although no special precautions were reported by the authors.

During the late 1980s, researchers at a Japanese pharmaceutical company used acidic *O*-acylations of L-DOPA in organic solvents for the preparation of a number of medicinally relevant L-DOPA derivatives [37–38]. A method based on HClO_4_ in organic solvents, very similar to that of Huang, Kimura, Bawarshi-Nassar and Hussain for tyrosine (Scheme 8), was used for an assortment of *O*-acylations of L-DOPA [37]. L-DOPA, in a mixture of aqueous HClO_4_ (70%) and either THF, dioxane or EtOAc, was mono-*O*-acylated with the acyl chlorides pivaloyl chloride, 1-methylcyclopropanecarbonyl chloride, cyclopropanecarbonyl chloride, valeryl chloride, 3,3-dimethylbutyryl chloride, octanoyl chloride, palmitoyl chloride, dodecanoyl chloride, benzoyl chloride and phenylacetyl chloride to the corresponding 4-*O*-acylated L-DOPA derivatives. In a second report, an impressive example, where 60 g of L-DOPA is *O*-acylated with pivaloyl chloride in 300 mL of CF_3_CO_2_H (keeping the temperature below 0 °C), was reported [38] – a testimony to the robustness and simplicity of acidic *O*-acylation procedures.

#### Resurrection and improvement of chemoselective *O*-acylation methodologies by the advent of asymmetric organocatalysis

In the previous section, the developmental history of acidic acylation methodologies for chemoselective *O*-acylation of hydroxyamino acids has been chronicled, from its inception in the early 1940s until the 1990s. In particular, the identification and application of suitable reaction media for such acidic acylations have been emphasized. While it is true that a number of convenient techniques for chemoselective *O*-acylations were developed during this time period, the application of these techniques nevertheless seems to have been strictly confined to biologically oriented chemistry, as a set of specialized techniques, useful for certain applications in peptide chemistry or in the synthesis of medicinal compounds from tyrosine or L-DOPA, besides, of course, the preparation and study of such *O*-acylated amino acids in itself. The employment of acidic *O*-acylations of hydroxyamino acids as a tool for such purposes would continue to flourish, and has to a certain extent remained to do so, for instance as a method for chemoselective *O*- and *S*-palmitoylation of peptides [39], as reported in 1999 (using palmitoyl chloride in CF_3_CO_2_H) or in the synthesis of *O*-lauroyl, *O*-myristoyl and *O*-phenylacetyl derivatives of DL-serine and threo-DL-phenylserine, as reported in 2003 (using the acyl chlorides in CF_3_CO_2_H), derivatives that displayed fungicidal activity [40]. However, by and large, the synthesis of amino acid or amino alcohol derivatives has relied on traditional protective group chemistry, much due to the pervasive role of flash column chromatography in modern organic synthesis.

The revitalization of enantioselective organocatalysis during the early 2000s [41], with a number of the most important organocatalysts ultimately descending from chiral amino acids, opened for new opportunities in the application of chemoselective *O*-acylation reactions as a toolkit for the preparation of amino acid-derived catalytic species on a large scale. Still, during the earliest period of this renewed interest in enantioselective organocatalysis, efforts by chemists were mainly directed towards the discovery of new catalytic principles or reaction types that could exploit a given catalytic concept. Then, during the second half of the first decade of revived organocatalysis, the more mature forms of organocatalysis were investigated and further developed in the context of concepts such as immobilized organocatalysts, water-compatible or water-dependent catalytic systems and finally nanoparticulate catalytic systems [41]. This eventually dictated a renewed interest in acidic *O*-acylation methodologies and set in motion events that would result in enhanced procedures.

#### Chemoselective *O*-acylation of hydroxyproline in fortified trifluoroacetic acid solutions

Proline catalysis was a cornerstone of early research into enantioselective organocatalysis [41]. For the preparation of solid-supported proline catalysts, the use of the naturally occurring and inexpensive hydroxyamino acid *trans*-4-hydroxy-L-proline is attractive due to its convenient 4-hydroxy group, a functionality that is ideally placed to function as a handle for further linkage to material supports. The field of such polymer and mesoporous material-supported organocatalysis has now been thoroughly analysed and documented through a number of recent reviews (an assortment of reviews prior to 2010 can be located in the stated references) [42–45].

**Scheme 9:**
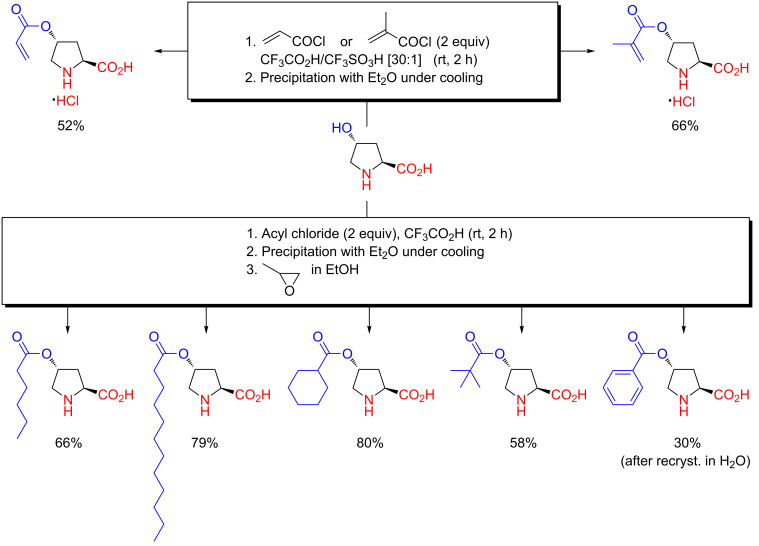
Preparation of proline amphiphiles or acrylic proline monomers (for macromolecular synthesis) by chemoselective *O*-acylation of hydroxyproline in CF_3_CO_2_H or CF_3_CO_2_H–CF_3_SO_3_H as reported in 2009 [46–47].

In 2009, Kristensen (the author of this text), Hansen and Hansen published an enhanced version of an acidic *O*-acylation that was used for the preparation of a variety of proline polymers and proline amphiphiles, on large scale, from hydroxyproline (Scheme 9) [46]. The work was later extended into a generalized system for polymeric immobilization of proline, prolineamide, diarylprolinol and imidazolidinone chiral organocatalysts by copolymerization of a set of acrylic precursors that were all prepared using an acidic *O*-acylation as the key step (detailed later) [47–48]. These disclosures apparently initiated and stimulated the widespread application of acidic *O*-acylation for the preparation of chiral organocatalysts that emerged in the years afterwards.

The fundamental motive for the development of an enhanced *O*-acylation procedure for hydroxyproline was the need to prepare the *O*-acryloyl and *O*-methacryloyl derivatives of hydroxyproline in large quantities. These side-chain derivatives, unlike those of other amino acids (serine, tyrosine, lysine and ornithine), were at this point entirely unknown in the literature. When the work leading up to these disclosures was initiated in 2006 and 2007, only the 1964 report by Wilchek and Patchornik on the *O*-acetylation of hydroxyamino acids with AcCl in HCl-AcOH-H_2_O (Scheme 4) and the 1983 report by Kawasaki and Komai on *O*-acylations of hydroxyproline in MeSO_3_H (Scheme 6) were known to the authors [20,34,46]. Use of copper chelate protective chemistry had failed, as had the first *O*-acylation attempts using acryloyl chloride in HCl–acrylic acid–H_2_O, in analogy with the known *O*-acetylation method of Wilchek and Patchornik [20]. As a complicating factor, unlike other side-chain derivatives of hydroxyamino acids, the *O*-acrylic derivatives are powerful acceptors for conjugate addition and must be kept strictly in salt form to avoid decomposition through self-addition. The problem is exacerbated in proline derivatives due to the excellent nucleophilicity of this particular amine scaffold. After a disclosure containing two *O*-acylations of serine in CF_3_CO_2_H with propionyl chloride and butyryl chloride was discovered, more or less by chance, in the patent literature (in what was then a patent application) [49], experimentations with this acylation medium begun, ignorant of the 1972 work of Previero, Barry and Coletti-Previero [27].

An acidic *O*-acylation of hydroxyproline with acryloyl chloride was possible but somewhat sluggish since this acyl chloride is considerably less reactive than the regular, saturated aliphatic acyl chlorides. The acylating activity of the medium was then fortified by the addition of trifluoromethanesulfonic acid (CF_3_SO_3_H), one of the strongest of all acids known (pK_a_ ≈ −5.5), comparable to, or exceeding perchloric and fluorosulfonic acid (although the ordering of acidities is highly dependent on the solvent medium) [29–30,50–51]. First prepared in 1954 [52], CF_3_SO_3_H (bp. 162 °C, *d* = 1.70 g/mL at 25 °C) exhibits excellent thermal, hydrolytic and oxidative/reductive stability, is miscible with both water and a wide variety of organic solvents and is not prone to fluoride anion generation [29,50]. The acylating power of the medium could thus easily be adjusted by varying the CF_3_CO_2_H/CF_3_SO_3_H ratio [46].

Hundreds of grams of proline amphiphiles and acrylic proline monomers were prepared by chemoselective *O*-acylation of hydroxyproline with saturated, aliphatic acyl chlorides in CF_3_CO_2_H (>20 g scale) or acryloyl/methacryloyl chloride in CF_3_CO_2_H–CF_3_SO_3_H (>30 g scale) (Scheme 9) [46–47]. The products were crystallized directly as their hydrochloride salts from the reaction mixture by addition of Et_2_O. The proline amphiphiles were liberated from their hydrochloride salts using propylene oxide in lower alcohols [46]. The acrylic derivatives, however, must be kept in salt form to avoid decomposition by conjugate addition. Acylation of hydroxyproline with aliphatic acyl chlorides in CF_3_CO_2_H gave a near quantitative yield of product (although the total yield was eroded by the subsequent recrystallizations), but yields were more moderate when using (meth)acryloyl chlorides.

As mentioned in passing earlier, the seminal publication by Previero, Barry and Coletti-Previero in 1972 on *O*-acylations of hydroxyamino acids in CF_3_CO_2_H does not, for unknown reasons, include hydroxyproline among the test substrates [27], possibly making the acylations summarized in Scheme 9 the first reported examples of such in the literature, at least to the best of this author’s knowledge [46–47]. In addition to the hydroxyproline derivatives depicted in Scheme 9, acylation of hydroxyproline with 2-methacryloyloxyethylsuccinoyl chloride in neat CF_3_CO_2_H could directly furnish an acrylic hydroxyproline monomer equipped with a linker segment [47]. This was later exploited by others (detailed later). The stability of hydroxyproline in the strongly acidic medium was found to be reassuring, substantiating the 1972 work by Previero, Barry and Coletti-Previero, detailed previously, regarding the stability of most of the common amino acids under acidic, non-hydrolytic reaction conditions (Table 1). The acidic conditions also guard the unprotected amino acid moiety against racemization through protonation close to the chiral centre. Although it is possible to locate assertions in the literature to the possibility of isolating the trifluoroacetate salts after *O*-acylations with acyl chlorides in CF_3_CO_2_H, as reported for L-tyrosine with pivaloyl chloride (the identity of the salt was, however, not determined) [38], it is rather doubtful when taking into account that nearly all other reports on *O*-acylations in CF_3_CO_2_H specifically indicate isolation of the hydrochloride salts, provided that an acyl chloride has been used as the acylating reagent.

While the 2009 disclosures discussed above were the first to report the *O*-acryloyl and *O*-methacryloyl derivatives of hydroxyproline, they were indeed not the first to report preparation of an *O*-acrylic derivative of a hydroxyamino acid using acidic *O*-acylation. In 2003, Hayakawa and Nemoto prepared *O*-methacryloyl L-tyrosine hydrochloride by acylation of L-tyrosine with methacryloyl chloride in CF_3_CO_2_H [53]. Also here, the product was crystallized directly from the reaction mixture by addition of Et_2_O. Working with materials of interest within the field of dentistry, the researchers were inspired by the work of Previero, Barry and Coletti-Previero from 1972 and used a similar methodology on a tyrosine amide already during the 1980s. Yet again, the application of the acidic *O*-acylation methodology within such a specialized field as dental materials has perhaps rendered the work rather opaque to materials scientists in general. This is unfortunate, as use of the mostly outdated and cumbersome copper protective chemistry has continued to dominate in the literature for the preparation of side-chain acrylic derivatives of hydroxyamino acids [5–8].

#### Use of chemoselective *O*-acylation of hydroxyamino acids for preparation of amphiphilic organocatalysts

Following the first 2009 report on the chemoselective *O*-acylation of hydroxyproline in CF_3_CO_2_H solutions [46], taken together with the potential for preparation of amphiphilic organocatalysts on large scale, in a protective group-free and non-chromatographic manner, the use of acidic *O*-acylation of hydroxyamino acids within asymmetric organocatalysis picked up momentum quite rapidly.

**Scheme 10:**
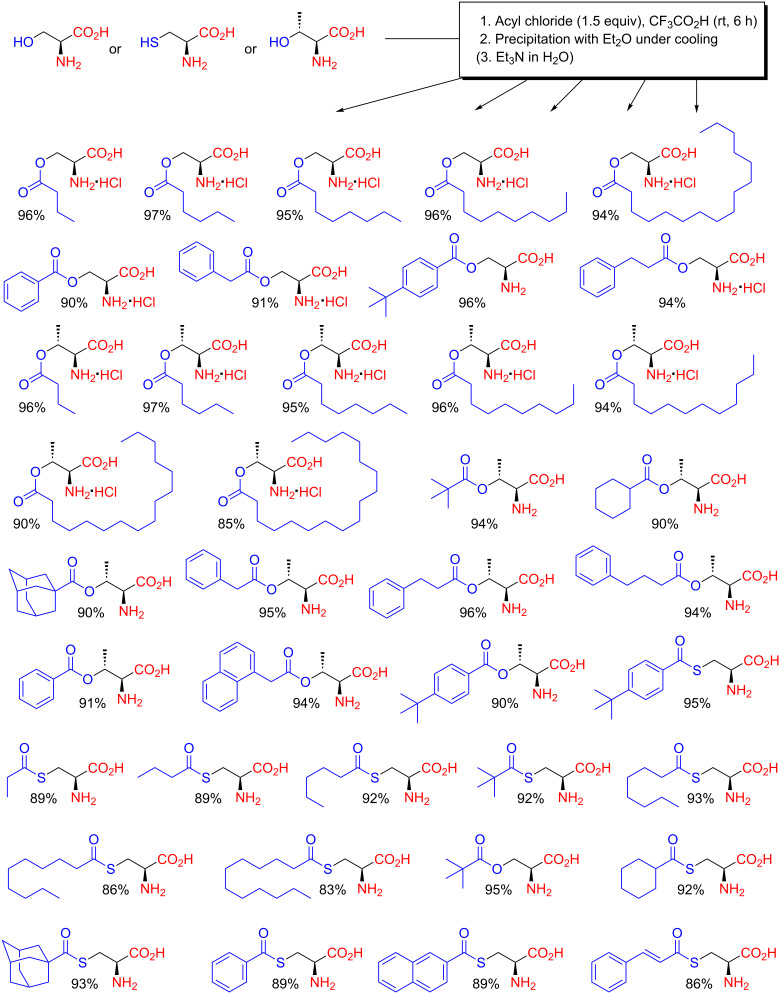
Preparation of amphiphilic organocatalysts from serine, threonine and cysteine by chemoselective *O*-acylation with acyl chlorides in CF_3_CO_2_H as reported in 2010–2012 [54–61].

Through a series of disclosures, during the time period from 2010 to 2012, Xiangkai Fu and co-workers detailed the preparation of amphiphilic organocatalysts from serine, threonine and cysteine by acidic *O*-acylation, as well as their use in asymmetric organocatalysis [54–61]. Chemoselective *O*-acylation of serine, threonine and cysteine in CF_3_CO_2_H with a range of acyl chlorides, followed by crystallization of the product directly from the reaction mixture by addition of Et_2_O, furnished a comprehensive collection of amphiphilic, chiral organocatalysts (Scheme 10). If necessary, the free amino acids were liberated by subsequent treatment of the hydrochloride salt with aqueous Et_3_N. Up to >100 g of a given amphiphilic organocatalyst could be prepared in a single acylation, making the convenience of the reaction evident [61]. The amphiphilic organocatalysts, often referred to as hydroxyamino acid (serine or threonine) surfactant organocatalysts by the authors, were then applied with success in asymmetric *anti*-Mannich reactions and asymmetric aldol reactions of ketones and aromatic aldehydes, in the presence of water [54–61]. Due to the nature of the reaction and ready availability of catalyst, these asymmetric reactions could be undertaken at the mole-scale (giving more than 100 g of product), with possibility of both catalyst recovery and reuse. The scalability of these reactions is first and foremost due to the convenience by which the catalysts could be prepared by acidic *O*-acylation.

**Scheme 11:**
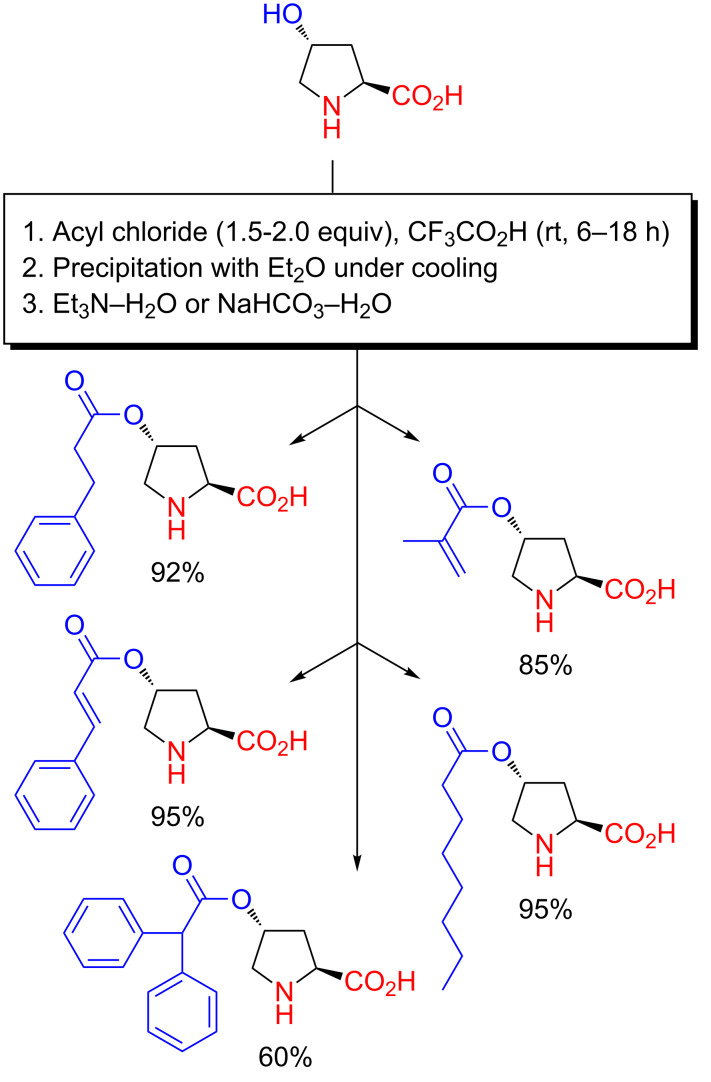
Preparation of amphiphilic proline organocatalysts by chemoselective *O*-acylation with acyl chlorides in CF_3_CO_2_H as reported in 2010 and 2012 [62–63].

Proline is perhaps the most central catalytic scaffold within organocatalysis, and in analogy to the exploitation of the serine, threonine and cysteine amphiphiles just discussed, the application of proline amphiphiles prepared through acidic *O*-acylation reactions has also been exhaustively investigated [62–67]. The same research group that reported the comprehensive assortment of amphiphilic organocatalysts depicted in Scheme 10 also employed the same procedures on hydroxyproline (Scheme 11) [62]. The *O*-diphenylacetoyl derivative was prepared using an analogous *O*-acylation procedure by Gruttadauria and co-workers in 2010 (Scheme 11) [63]. These compounds proved very efficient as organocatalysts in asymmetric aldol reactions in aqueous media. Even catalyst recovery and reuse (up to seven cycles) for aldol reactions at large scale (200 mmol of substrate) were possible [62].

**Scheme 12:**
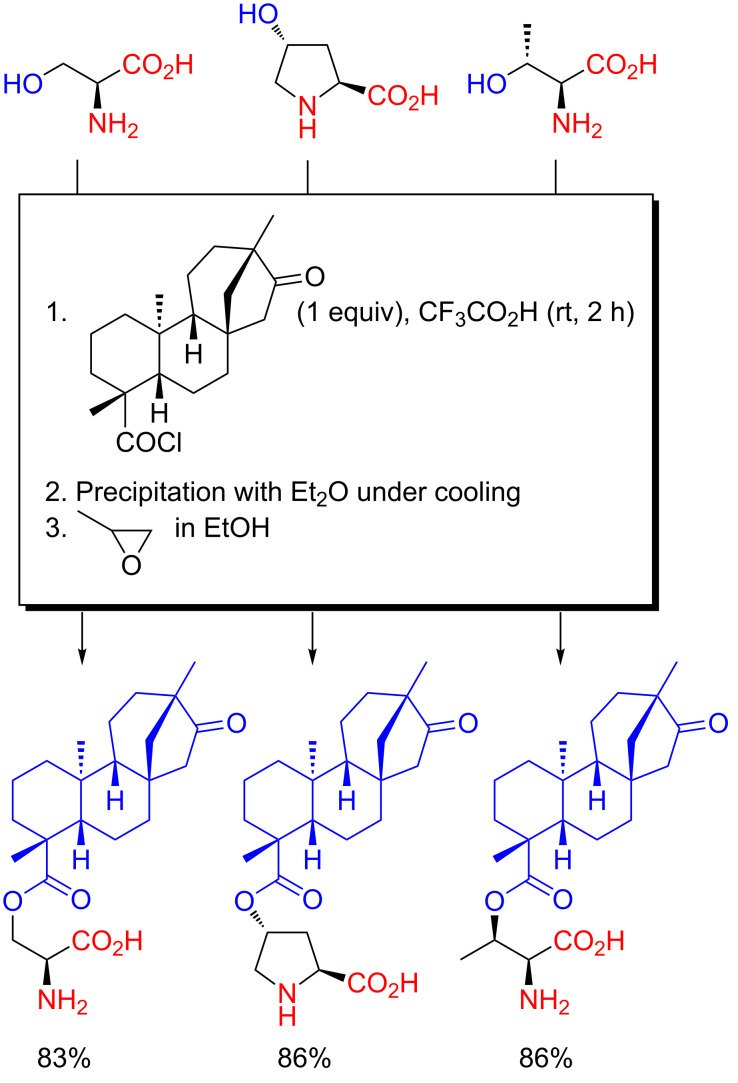
Amphiphilic organocatalysts prepared from hydroxyamino acids and isosteviol by chemoselective *O*-acylation in CF_3_CO_2_H [65–67].

A particularly bulky type of hydroxyamino acid amphiphiles has recently been reported by Tao and co-workers (Scheme 12) [65–67]. The tetracyclic diterpenoid isosteviol was converted to its corresponding acid chloride with SOCl_2_. The acid chloride was subsequently used directly in acidic *O*-acylation reactions of hydroxyproline, serine and threonine in CF_3_CO_2_H at gram-scale. The product hydrochlorides were converted to free amino acids with propylene oxide in EtOH. Optionally, the ketone functionality of the isosteviol moiety could be reduced to the alcohol with NaBH_4_–EtOH, thus furnishing a second set of modified amphiphilic organocatalysts. These isosteviol-modified hydroxyamino acid organocatalysts were tested with success in asymmetric aldol reactions, α-aminoxylation reactions and three-component Mannich reactions under aqueous conditions [65–67].

#### Use of chemoselective *O*-acylation of hydroxyproline for preparation of polymeric organocatalysts

The last couple of years have witnessed an interesting proliferation in the use of chemoselective *O*-acylation of hydroxyproline with acrylic acyl chlorides in CF_3_CO_2_H solution as a key step in the synthesis of acrylic hydroxyproline monomers [46–48,68–71]. Monomers originating from acidic *O*-acylation reactions can be used as precursors for the synthesis of macromolecular entities in the form of proline-functionalized high-load homopolymers by solution copolymerization [46–47], crosslinked polymer microspheres by dispersion copolymerization [47], crosslinked polymer beads by suspension copolymerization [47–48], catalytic core–shell micelles (nanoreactors) [68], hydrophobic nanogel particles by emulsion polymerization [69], magnetic core-shell nanoparticles (with magnetite cores and polyacrylate shells) [70], and thermoresponsive block copolymers [71]. Such catalytic systems can exhibit excellent organocatalytic activity under aqueous conditions, and many of them can be recycled and reused. A rather unique property of such polymers prepared from side-chain-linked hydroxyproline or other hydroxyamino acids is their zwitterionic character, thereby separating them from more conventional amino acid acrylates prepared from the *N*-acylated amino acids. In the future, more resources could favourably be directed, by polymer scientists, towards the study of such zwitterionic polymers.

Also, many catalytically active macromolecular systems on the basis of proline have been produced from monomers that have been prepared using more traditional protective group chemistry and they are therefore not detailed herein, but many of these can conceivably be more effortlessly accessed from monomers prepared using acidic *O*-acylations.

**Scheme 13:**
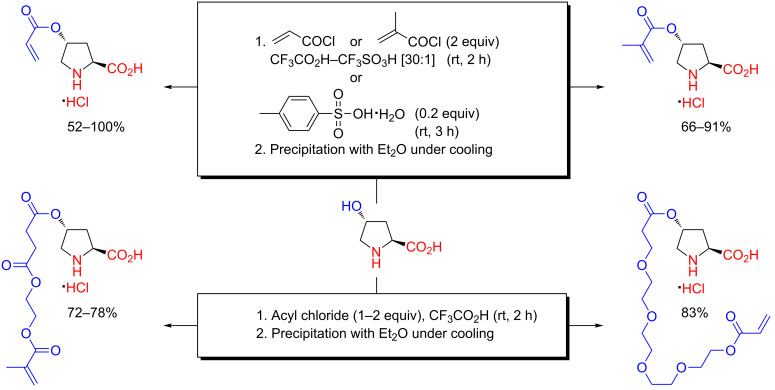
Preparation of acrylic proline precursors for polymeric organocatalysts by chemoselective *O*-acylation of hydroxyproline in CF_3_CO_2_H solutions [46–47,68–71].

Proline-functionalized macromolecular systems prepared from acrylic precursors that have been accessed through acidic *O*-acylation reactions have so far been based on four main acrylic hydroxyproline derivatives (Scheme 13) [46–47,68–71]. Acylation of hydroxyproline in CF_3_CO_2_H–CF_3_SO_3_H with acryloyl or methacryloyl chloride gives the corresponding acrylic ester hydrochlorides usually in moderate to good yields [46–47], although a quantitative yield of the acrylate has been reported [70]. However, an interesting modification has been reported by O’Reilly and co-workers wherein the CF_3_SO_3_H in the acylating medium was substituted with a small quantity of *p*-toluenesulfonic acid monohydrate, thereby allowing preparation of the *O*-methacryloyl ester of hydroxyproline as hydrochloride salts in near quantitative yield (Scheme 13) [68–69]. Acrylic proline monomers equipped with a linker segment can be prepared through acylations using long-chained acrylic acyl chlorides (Scheme 13) [47,70]. The commercially available 2-methacryloyloxyethylsuccinic acid is a particularly convenient starting point [47,70].

**Scheme 14:**
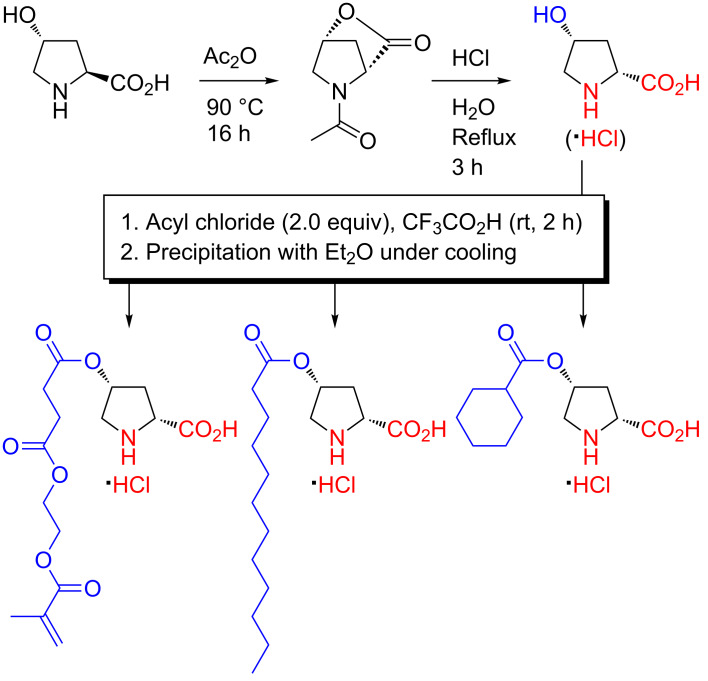
Conversion of *trans*-4-hydroxy-L-proline to *cis*-4-hydroxy-D-proline·HCl and subsequent chemoselective *O*-acylation in CF_3_CO_2_H [48,74].

A potential obstacle to the application of organocatalytic systems based on naturally occurring *trans*-4-hydroxy-L-proline as the catalytic moiety is how to access both series of enantiomeric products, since the enantiomer of the naturally occurring hydroxyproline is not easily available. Fortunately, a partial, and simple, solution is available through the facile conversion of *trans*-4-hydroxy-L-proline to its diastereomer *cis*-4-hydroxy-D-proline, a conversion that has been studied and modified for more than 60 years [72]. Treatment of *trans*-4-hydroxy-L-proline with acetic anhydride under heating, followed by hydrolysis in dilute hydrochloric acid, gives *cis*-4-hydroxy-D-proline·HCl via an *N*-acetylated lactone [73], a product that can be acylated directly in the same manner as the natural hydroxyproline (Scheme 14) [48,74]. In addition to acrylic proline monomers [48], the *O*-acylation of *cis*-4-hydroxy-D-proline (albeit not the hydrochloride salt in this case) has also been employed as a key step in the preparation of ligands for new chiral Rh(II) catalysts (Scheme 14) [74].

### Extension of chemoselective acidic *O*-acylation reactions to other substrates and comparison with other available methodologies

In the final section of this review, attention will be directed at the extension of the methodology that, up to this point, has been chronicled purely in the context of the acylation of hydroxyamino acids, to describe the *O*-acylation of amino alcohol structural motifs in general, as well as to the closely connected *O*-sulfation reactions. Finally, a brief comparison will be made between the acidic *O*-acylation techniques, treated so far, with other available methodologies for chemoselective *O*-acylation, some of which have emerged recently.

#### Chemoselective *O*-acylation of amino alcohols under acidic conditions

Alongside the development of a chemoselective acidic *O*-acylation of hydroxyamino acids, analogous techniques have been applied to amino alcohols. However, as the class of amino alcohols in the broadest sense encompasses such a vast collection of compounds, no singular, unifying methodology may hope to succeed, to the same extent, as with the small number of naturally occurring hydroxyamino acids. Merely, a collection of some prominent examples can be highlighted. In the same manner as for acidic *O*-acylation of hydroxyamino acids, the advent of asymmetric organocatalysis has also done much to promote the application of such methods for amino alcohols.

As a matter of historic fact, the chemoselectivity issues associated with the competitive, partial acylation of alcohols in the presence of amines seem first to have been encountered as part of chemical investigations into the nature of physiologically important aromatic amino alcohols such as ephedrine, pseudoephedrine, adrenaline, adrenalone and synephrine [75–85]. Some of this work took place even before the first analogous work on hydroxyamino acids by Sakami and Toennies [9–10], but the exact starting point is hard to pin down because there is a natural delay between the execution of experiments and the attainment of proper chemical understanding of what had taken place during those experiments. The literature of such early experimentation on amino alcohol natural products is too voluminous to justify any exhaustive listing in this work, thus for the purposes herein, it will suffice to point out that the outcome of acetylation reactions on such amino alcohols varied greatly depending on the acidity or alkalinity of the reaction medium.

**Scheme 15:**
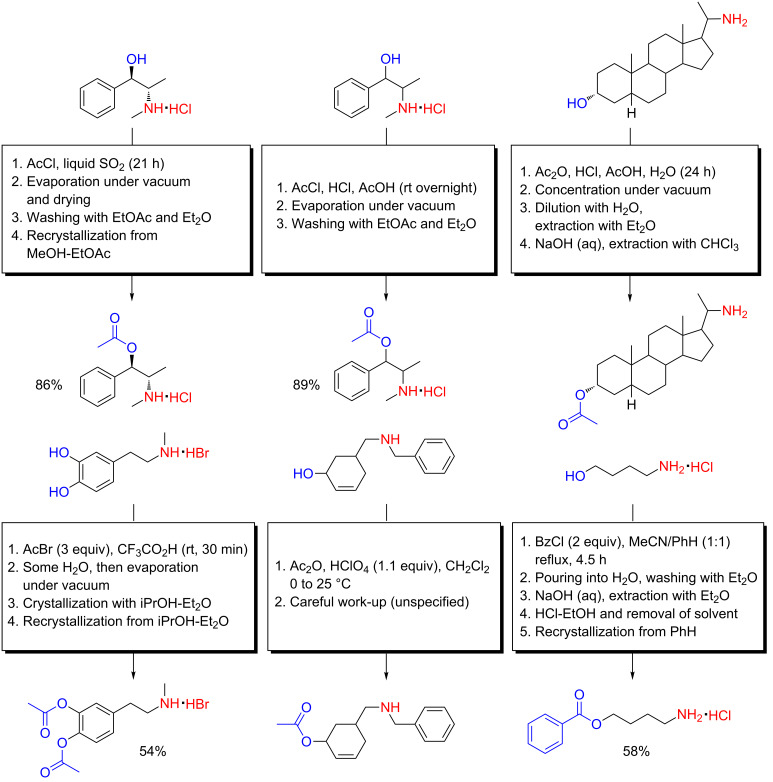
Some examples of chemoselective *O*-acylation of amino alcohols under acidic reaction conditions reported in the literature [77,86–88,90].

In a previous section, we were acquainted with the method by which Bretschneider and Biemann *O*-acetylated L-tyrosine under acidic conditions (Scheme 2) [13], a method referred to by Bretschneider as salt acylation. Salt acylation was indeed first developed through his comprehensive work on natural product amino alcohols during the 1940s [76–85]. Quite exotic reaction conditions were investigated for such salt acylations, as exemplified through the chemoselective *O*-acetylation of ephedrine hydrochloride with acetyl chloride in liquid SO_2_ (Scheme 15) [77]. More convenient was the *O*-acetylation of racemic ephedrine hydrochloride with acetyl chloride in HCl-saturated glacial acetic acid (Scheme 15) [77], the method that was later adopted for the *O*-acetylation of L-tyrosine (Scheme 2) [13]. A similar method, in which a steroidal amino alcohol was acetylated with acetic anhydride in a mixture of glacial acetic acid and concentrated hydrochloric acid, was reported by Robinson, Milewich and Hofer in 1966 (Scheme 15) [86]. This procedure bears resemblance to the method of Wilchek and Patchornik from 1964 by which hydroxyamino acids were *O*-acetylated under the same conditions (Scheme 4) [20].

A particularly interesting example of an acidic *O*-acetylation of an amino alcohol was reported by Borgman, McPhillips, Stitzel and Goodman in 1973 [87]. Adapting the 1972 work by Previero, Barry and Coletti-Previero on hydroxyamino acids [27], a medicinally relevant catecholamine (as its hydrobromide salt) was chemoselectively *O*,*O’*-diacetylated at gram-scale with acetyl bromide in anhydrous CF_3_CO_2_H (Scheme 15). Additionally, this is also a rare example of the use of an acyl bromide in an acidic *O*-acylation reaction.

Individual examples of acidic *O*-acylation reactions are scattered throughout the literature. In 1976, Trost and Genêt used a careful *O*-acetylation of an amino alcohol alkaloid intermediate with acetic anhydride in CH_2_Cl_2_ acidified with HClO_4_ as part of a total synthesis (Scheme 15) [88]. In the footsteps of Bretschneider, chemoselective *O*-acetylation of natural product amino alcohols also inspired others to employ similar methods for hydroxyamino acids. The *O*-acylation of L-tyrosine in acidified EtOAc solution reported by Huang, Kimura, Bawarshi-Nassar and Hussain in 1985 (Scheme 8) was actually the result of previous work in the same research group from 1976, where the method had been used in the preparation of adrenaline esters [89]. Dissimilar to the developmental work reported for acidic *O*-acylation of hydroxyamino acids, the analogous work on amino alcohols undeniably gives the impression of being largely constrained to acetylations.

Frustrated by an alleged lack of generality in available procedures for acidic *O*-acylation of amino alcohols, Luh and Chong reported a method for selective aromatic esterification of amino alcohols in 1978, consisting of acylations of an amino alcohol hydrochloride with (predominantly aromatic) acyl chlorides in acetonitrile–benzene (Scheme 15) [90]. The reaction was found to be very solvent dependent, and a reduction of basicity of the solvent system was beneficial. Selective esterification was much improved in acetonitrile compared to HMPA or THF and admixture of benzene to the acetonitrile resulted in further improvement. Amino alcohols endowed with the possibility of facile intermolecular proton transfers (3-aminopropanol, 4-hydroxypiperidine) gave mainly the unwanted amide product [90].

A few relevant disclosures with regards to *O*-acylation of amino alcohols continued to surface through the 1980s and up to present times [91–92]. Among these, Kihara, Shin, Ohga and Takata reported in 2001, a selective *O*-acylation of an amino alcohol in CH_2_Cl_2_ with a bulky acid anhydride in the presence of CF_3_SO_3_H as part of a rotaxane synthesis [92]. The reaction mixture contained only a slight excess of acid, and use of powerful CF_3_SO_3_H was necessary in order to both protect amine by protonation and catalyze the esterification. In the presence of CF_3_CO_2_H, only trace amounts of the amide could be detected and no reaction took place when MeSO_3_H was used (catalysis of the esterification was inadequate). However, it is important to keep in mind that the relevant aromatic acylating agents employed are quite unreactive, and use of a slight stoichiometric excess of acid (relative to amines present) gives rise to very different reaction conditions compared to the *O*-acylations taking place in neat CF_3_CO_2_H or MeSO_3_H that have been most relevant for the works covered in this review.

The exponential growth of asymmetric organocatalysis in the first decade of the 21st century was an ideal opportunity for the resurgence of acidic *O*-acylation methodologies, not only for hydroxyamino acid-derived organocatalysts, but also for associated amino alcohol structural motifs. This has been of particular relevance in the case of polymer-supported enamine and iminium organocatalysts [48,93–94].

**Scheme 16:**
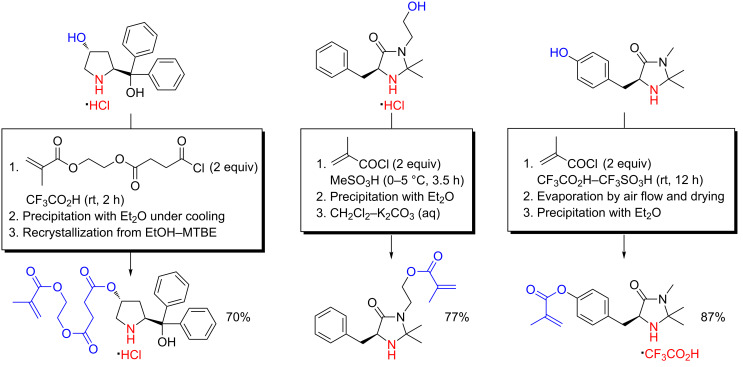
An assembly of chiral acrylic building blocks useful in the synthesis of polymer-supported diphenylprolinol and imidazolidinone organocatalysts using chemoselective *O*-acylation under acidic reaction conditions as the key step [48,93–94].

Building on their work on *O*-acylation of hydroxyproline with acrylic acyl chlorides in CF_3_CO_2_H medium as a method for proficient synthesis of building blocks for organocatalytic macromolecular networks [46–47], Kristensen, Vestli, Jakobsen, Hansen and Hansen further exploited this concept as a key step in the large-scale and non-chromatographic synthesis of polymer-supported diphenylprolinol and imidazolidinone organocatalysts (Scheme 16) [48]. Hydroxydiphenylprolinol hydrochloride could be *O*-acylated selectively at the secondary alcohol, in the presence of both an amine and tertiary alcohol, with an acrylic acyl chloride in CF_3_CO_2_H at more than 30 g scale, furnishing a crystalline reaction product (Scheme 16). Further on, a MacMillan imidazolidinone intermediate was *O*-acylated on multigram-scale with methacryloyl chloride in MeSO_3_H to an oily methanesulfonate product (Scheme 16). These two intermediates were subsequently used as monomeric building blocks in the synthesis of microporous polymer beads through suspension copolymerization with suitable acrylic comonomers [48]. Together with building blocks derived from the *O*-acylation of hydroxyproline with acrylic acyl chlorides, these intermediates formed the foundation for a quite general approach to polymer-supported chiral enamine and iminium organocatalysts.

A similar approach to polymer-supported organocatalysts was pursued by O’Reilly and co-workers for the MacMillan imidazolidinone organocatalyst [93–94]. Using tyrosine instead of phenylalanine as the starting material for the imidazolidinone synthesis, a phenolic functionality amenable to acylation was available. Acylation with methacryloyl chloride in CF_3_CO_2_H–CF_3_SO_3_H, followed by a careful work-up, provided an acrylic MacMillan precursor suitable for macromolecular synthesis of functional polymers (Scheme 16). This precursor was used for reversible addition-fragmentation chain transfer (RAFT) copolymerization reactions with diethylene glycol methyl ether methacrylate and for preparation of crosslinked nanogels by oil-in-water emulsion polymerization and core-shell type nanogels by seeded polymerization [93–94]. The catalytic activities of the catalysts were evaluated in asymmetric Diels–Alder reactions between cyclopentadiene and olefins.

**Scheme 17:**
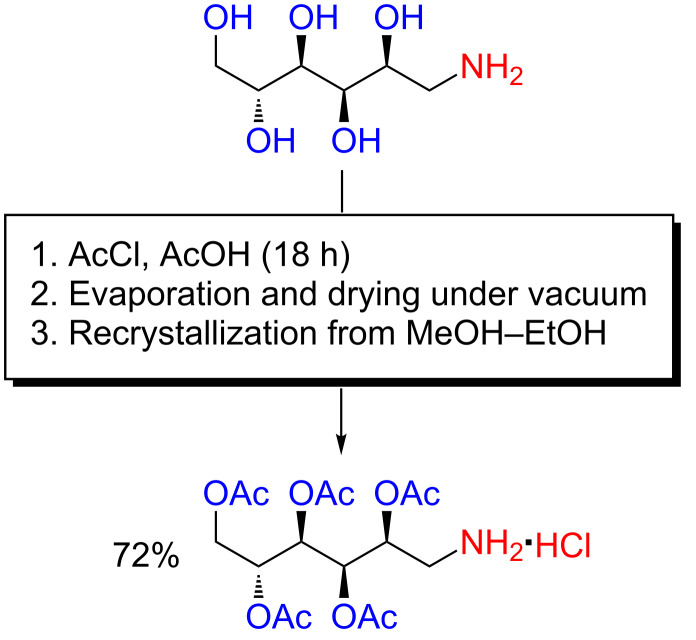
The chemoselective pentaacetylation of D-glucamine under acidic reaction conditions [95].

The employment of acidic reaction conditions for chemoselective *O*-acylation of amino alcohols has proven useful in a number of applications, as has been amply demonstrated already, also outside the realm of organocatalysis. A particularly striking example was reported by Paik, Tapriyal, Enick and Hamilton in 2007, wherein D-glucamine, a molecule with no less than five hydroxy groups and one primary amine group, was chemoselectively pentaacetylated at gram-scale in glacial acetic acid with acetyl chloride – reaction conditions the reader, by now, will recognize as quite classic for acidic *O*-acylations, although the absence of a strong acid is unusual (Scheme 17) [95]. The reaction product was an intermediate in the preparation of highly CO_2_-soluble bisureas of relevance to the use of supercritical CO_2_ as solvent. It should be noted that CH_2_Cl_2_ has, for some reason, been stated as a reaction solvent in one of the reaction schemes in the relevant publication, but not in the appurtenant experimental procedure provided in the supporting information [95]. The role of such a non-polar reaction solvent in this particular transformation is unclear and perhaps erroneous, and it is therefore not included in Scheme 17.

#### Chemoselective *O*-sulfation reactions under acidic reaction conditions

For obvious reasons, the possible extension of the acidic acylation procedures described up to this point, using carboxylic acid derivatives, to analogous reactions using sulfur oxyacid derivatives has been investigated to some extent. However, the methodology does not seem to be directly applicable to *O*-sulfonylation reactions, judging from the apparent lack of such examples in the literature of chemistry. Still, an important exception is the simple chemoselective *O*-sulfation of hydroxyamino acids to their corresponding sulfate esters. In biochemistry, tyrosine sulfation, the enzymatic addition of a sulfate ester to a tyrosine residue of a protein (posttranslational modification) is an important concept. Therefore, the establishment of suitable reaction conditions for the preparation of the sulfate esters of hydroxyamino acids has been of palpable interest for some time.

Chemoselective *O*-sulfation of serine, threonine, hydroxyproline and tyrosine can be achieved through simple treatment of the hydroxyamino acid with cold concentrated H_2_SO_4_ as reported by Reitz, Ferrel, Fraenkel-Conrat and Olcott in 1946 [96]. The products were separated through addition of precipitating solvents such as Et_2_O or methyl ethyl ketone and then recrystallized. This method has later been slightly modified by others [97–98].

In 1979, the same research group that during the early 1970s pioneered the acidic *O*-acylation reactions of hydroxyamino acids in anhydrous CF_3_CO_2_H, reported a chemoselective *O*-sulfation of L-serine and L-threonine with ClSO_3_H in anhydrous CF_3_CO_2_H (about 20 min reaction time at room temperature) [27,99]. The reaction takes place without racemization, and the crude product was separated by addition of Et_2_O and recrystallized from aqueous acetone. DL-Tyrosine was converted into tyrosine 3-sulfonic acid under the same reaction conditions. The conversion could also be affected, albeit more slowly, through use of H_2_SO_4_ as the reagent. Exactly as in their previous work on acylation reactions [27], the research group tested the stability of a number of the common amino acids under the reaction conditions, recovering nearly all of them (except tryptophan) in quantitative yields [99]. Yet again, anhydrous CF_3_CO_2_H was identified as a convenient medium in which to transform hydroxyamino acids under acidic reaction conditions. The same *O*-sulfation methodology has been tested successfully on peptides, converting them to strongly acidic derivatives [99]. Others have later employed the method on both hydroxyamino acids and peptides as part of their studies [100–101].

An interesting area of application for chemoselective *O*-sulfation procedures, which has emerged recently within organic synthesis, is the preparation of silica-supported hydroxyproline 4-sulfate ester by simple treatment of hydroxyproline with neat ClSO_3_H, followed by addition of silica gel [102]. This material can be a useful solid-supported catalyst for some reactions.

#### Comparison of acidic *O*-acylations with other available methods for chemoselective *O*-acylation of amino alcohols

A small number of alternative methods for chemoselective *O*-acylation of amino alcohols have quite recently emerged alongside the classic types of acidic acylations considered up to this point. These have attracted widespread interest, a testimony to the perceived difficulty of the fundamental nature of nucleophilic chemoselectivity issues associated with amines in the presence of alcohols. In lieu of the obvious promotion of acidic acylation methodologies through this work, it is only fitting that a brief comparison with other methods is presented.

As a starting point, given the obvious practicality of acidic *O*-acylation for robust, large-scale and non-chromatographic preparation of a number of esters of hydroxyamino acids and amino alcohols, as amply documented through this review, the rather terse mention of said techniques as part of extensive reviews on the relevant chemoselectivity issues can only be described as discomforting, quite lacking in both scope and historical context [103–105]. The recent disclosures to be discussed below are unfortunately also quite contextually scant, taking into account the long history of alcohol-amine chemoselectivity studies. Hopefully, the present review will be perceived as timely and may help to amend the prevailing situation.

As for other methods for chemoselective *O*-acylation of amino alcohols, a detailed and comprehensive historical assessment will not be attempted and only some of the more recent, high-profiled disclosures will be emphasized. Biochemical methods based on lipases in organic solvents for chemoselective acylation of peptides have been developed for a number of years [106]. Also within organocatalysis, which has been an important arena for the advancement of acidic *O*-acylation techniques, chemoselective lipase-catalyzed esterification of hydroxyproline with methacrylic acid has been reported [107]. However, the practicality of this biochemical method appears limited in a preparative context.

A particularly high-profiled method for chemoselective acylation of alcohols in the presence of amines was reported by Ohshima, Iwasaki, Maegawa, Yoshiyama and Mashima in 2008 [108]. Using the customized tetranuclear zinc cluster Zn_4_(OCOCF_3_)_6_O, alcohols could be selectively acylated with methyl esters in the presence of amines using refluxing iPr_2_O as the reaction solvent. The method is to some extent hampered by a number of complications, such as the use of a customized catalyst, long reaction times (18–24 h), a hazardous reaction solvent, non-proven scalability and the use of chromatographic purification. However, its employment of reasonably mild acylating reagents (esters) and reaction conditions is attractive.

Another recent method for the chemoselective acylation of alcohols in the presence of amines, that is particularly worthy of mention, is the *N*-heterocyclic carbene (NHC)-mediated oxidative, chemoselective esterification of amino alcohols with cinnamaldehyde reported by Studer and co-workers from 2010 onwards [109–110]. Oxidatively generated acylazolium species is the acylating reagent and the NHC serves a dual role as a cooperative catalyst, both for the generation of the acylazolium species as well as the activation of the alcohol in the subsequent acylation step. However, for amino alcohols, the substrate should not have the possibility of intramolecular acyl transfer after esterification, severely restricting the applicability of the reaction. Again, the utilization of a number of specialized reagents could limit the widespread adoption of this methodology. In addition, this process has yet to be proven effective in large-scale preparations, which may prevent its use in related fields such as materials science.

All in all, it is safe to assume that there remains a huge potential for improvements regarding the development of convenient, broadly applicable and scalable procedures for the chemoselective acylation of alcohols in the presence of amine functionalities. For the time being though, the acidic acylation methodologies chronicled herein, stand their ground quite well when challenged by more sophisticated synthetic machinery. However, such new methods are much needed due to the obvious shortcoming of acidic acylation methods, namely its uselessness when dealing with substrates sensitive to acidic reaction conditions.

## Conclusion

Chemists long have had a profound fascination for chemoselectivity – the problem of how to choose only one functional group, among several (near) equals in the course of a chemical transformation – something of obvious interest for a field focused on chemical bonding and chemical reactivity. Given the current advanced state of organic synthesis, it is perhaps surprising that such a conceptually simple problem as the classic alcohol–amine chemoselectivity issue continues to be one of the most vexing and annoying problems of the field. Synthetic detours using protective group chemistry remain the mainstay solution to this problem and recent progress in the application of CO_2_ as a temporary amine-protecting agent might perhaps be a useful such solution in some situations [111].

The simple use of acidic versus alkaline reaction conditions, whether aqueous or in organic solvents, as a strategy for controlling alcohol-amine chemoselectivity during acylations, is an old idea, stumbled upon in a more or less serendipitous manner and then eagerly investigated by chemists from (at least) the 1940s and onwards. Deceptively simple, the development of generalizable procedures with a useful scope has nonetheless challenged generations of chemists with a shrewd acumen for combining chemical reactivity and solubility. Unfortunately, as much of these pioneering studies took place in a biochemistry-oriented setting, the lessons learned have perhaps remained somewhat outside the venues most conducive to the mainstream community in organic synthesis. On the positive side, the last few years have witnessed a resurrection in the opportunities offered by acidic modes of acylation, broadening the opportunities for future improvements.

With regards to acidic *O*-acylation methods, just as for chemoselective methods in general, there is plenty of room for further advancements. In particular, new reaction media are needed, much because of the increased focus on renewable and environmentally acceptable solvent systems. Perhaps the best solvent for acidic *O*-acylations (at least for hydroxyamino acids), trifluoroacetic acid, cannot be said to fulfil such requirements. The use of less reactive acylating agents than acyl halides and carboxylic anhydrides should also be a priority in future endeavours. A noticeable peculiarity in the history of acidic *O*-acylations is the near absence of other acyl halides than acyl chlorides. Recently, the use of iodide as an activating agent for acyl chlorides in acylation reactions, presumably through a transient acyl iodide intermediate, has been reported, something which is perhaps applicable to acidic *O*-acylation reactions as well [112]. Finally, an important topic for more refined methods for acidic *O*-acylation methods is their relevance to regioselectivity issues, a topic that has barely been touched upon in this review.
